# Susceptibility Analysis in Several Mouse Strains Reveals Robust T-Cell Responses After *Mycoplasma pneumoniae* Infection in DBA/2 Mice

**DOI:** 10.3389/fcimb.2020.602453

**Published:** 2021-01-13

**Authors:** Shigeyuki Tamiya, Eisuke Yoshikawa, Koichiro Suzuki, Yasuo Yoshioka

**Affiliations:** ^1^ Laboratory of Nano-design for innovative drug development, Graduate School of Pharmaceutical Sciences, Osaka University, Osaka, Japan; ^2^ Vaccine Creation Group, BIKEN Innovative Vaccine Research Alliance Laboratories, Institute for Open and Transdisciplinary Research Initiatives, Osaka University, Osaka, Japan; ^3^ Vaccine Creation Group, BIKEN Innovative Vaccine Research Alliance Laboratories, Research Institute for Microbial Diseases, Osaka University, Osaka, Japan; ^4^ The Research Foundation for Microbial Diseases of Osaka University, Osaka, Japan; ^5^ Global Center for Medical Engineering and Informatics, Osaka University, Osaka, Japan

**Keywords:** infection, inflammation, mouse strains, *Mycoplasma pneumoniae*, neutrophils, Th cells

## Abstract

*Mycoplasma pneumoniae* (Mp) is a highly contagious respiratory pathogen responsible for human community-acquired pneumonia. The number of antibiotic-resistant Mp strains is increasing; therefore, to develop novel therapeutics, it is crucial to precisely understand the pathogenesis of mycoplasma pneumonia. Herein, we examined the susceptibility and response to Mp among eight inbred mouse strains. Following infection, the bacterial load in the bronchoalveolar lavage fluid (BALF) from DBA/2 mice was higher than that in the other tested strains such as BALB/c mice, which are frequently used in Mp research. In contrast, the numbers of CD45^+^ immune cells and neutrophils in BALF were comparable between BALB/c and DBA/2 mice, with lower numbers observed in C57BL/6J and CBA/N mice than in BALB/c mice. Among the tested strains, the BALF level of interleukin 12 subunit p40 was highest in DBA/2 mice; however, significant differences in other cytokines levels were not observed between BALB/c and DBA/2 mice. After Mp infection, Mp-specific Th1 and Th17 responses were significantly enhanced in DBA/2 mice when compared with BALB/c mice. Furthermore, prior infection with Mp increased the number of neutrophils in BALF after the reinfection of DBA/2 mice through an Mp-specific CD4^+^ T cell-dependent mechanism. Thus, DBA/2 may be an appropriate strain for evaluating Mp infection. Moreover, a comparison of responses revealed by various inbred mouse strains could be useful for elucidating the pathogenesis of Mycoplasma pneumonia.

## Introduction

*Mycoplasma pneumoniae* (Mp), a human mycoplasma species, is a common, highly contagious respiratory pathogen (Waites and Talkington, 2004; Waites et al., 2017). In children, Mp is responsible for a high percentage of community-acquired bacterial pneumonia (Waites et al., 2017). Mp infections persist for weeks extracellularly in the respiratory tract and induce the formation of chronic inflammatory lesions along the airways (Saraya et al., 2014). Tracheobronchitis is a particularly common clinical symptom (Waites and Talkington, 2004). Furthermore, an Mp infection can exacerbate other diseases and conditions, including asthma and myocarditis (Waites and Talkington, 2004).

Notably, Mp is resistant to inhibitors of cell wall synthesis as the bacteria lack a peptidoglycan layer; therefore, macrolide antibiotics are the recommended treatment for Mycoplasma pneumonia (Meyer Sauteur et al., 2016). However, macrolide-resistant Mp strains have emerged worldwide, possibly as a result of extensive macrolide usage (Meyer Sauteur et al., 2016). Despite efforts spanning decades to develop a safe and efficacious vaccine against Mp, a suitable vaccine is yet to be developed for humans.

The virulence of Mp is dependent on the ability of the bacteria to use its cytoadhesin molecules to attach to and move across ciliated epithelial cells in the respiratory tract *via* glycoprotein receptors containing terminal sialic acid residues, resulting in upper and lower airway infections (Waites and Talkington, 2004; Waites et al., 2017). After attachment, Mp induces inflammatory responses such as neutrophil infiltration and the secretion of cytokines and chemokines (Waites and Talkington, 2004; Waites et al., 2017). Notably, toll-like receptor 2 (TLR2) is essential for the inflammatory response toward Mp as it possesses membrane-bound lipoproteins that are TLR2 ligands (Shimizu, 2016).

Reportedly, lung injury has been observed in mycoplasma pneumonia and is caused by an excessive immune-inflammatory response against Mp as glucocorticoid and immunoglobulin treatments remain efficacious against refractory mycoplasma pneumonia (Tamura et al., 2008; Shan et al., 2017). In particular, neutrophils infiltrating the lung after Mp infection contribute to the lung-tissue damage observed in humans (Guo et al., 2015; Chen et al., 2016; Zhang et al., 2016a). In patients with refractory mycoplasma pneumonia, high numbers of neutrophils have been observed in the peripheral blood and bronchoalveolar lavage fluid (BALF) (Guo et al., 2015; Zhang et al., 2016a). In addition, Mp-specific T cells activated by Mp infection contribute to the inflammatory response in the lung (Dobbs et al., 2009). Nevertheless, the mechanism of T cell-mediated inflammation, including the characterization of the CD4^+^ T-cell subset related to neutrophil infiltration, remains largely undefined. Therefore, mechanisms underlying the inflammatory responses to Mp infections need to be elucidated.

Genetic factors are known to be crucial in regulating the severity of infectious diseases in mice and humans (Gruenheid and Gros, 2010; Chapman and Hill, 2012). However, it is difficult to clarify the relationship between phenotype and genotype in humans owing to experimental limitations such as cohort size and the impact of environmental factors. To address these issues, phylogenetically different inbred mouse strains have been widely used to determine differences in susceptibility to bacterial infections (De Simone et al., 2014; Spagnuolo et al., 2016; Lore et al., 2018). This approach has been successful because of the common genetic background of each strain, resulting in low variability in responses to infections. For example, several studies have used this approach to examine the impact of the host genetic background during respiratory infection by *Pseudomonas aeruginosa* in mice (Lore et al., 2018). With regard to Mp, it has been reported that the Mp bacterial load in BALF is higher in BALB/c mice than in C57BL/6 mice after Mp infection (Lai et al., 2010). Therefore, a comparison of responses to Mp infection from different mouse strains could be a useful means for examining inflammatory responses to Mp infections.

Herein, we conducted a susceptibility study comparing the Mp bacterial load and immune responses after infection in eight inbred mouse strains (A/J, BALB/c, C3H/HeJ, C3H/HeN, C57BL/6J, CBA/N, DBA/1, and DBA/2). Among these mouse strains, DBA/2 mice showed the highest bacterial load in the lung after Mp infection. Additionally, DBA/2 mice showed more robust Mp-specific Th1 and Th17 immune responses after Mp infection when compared with the responses observed in BALB/c mice. Furthermore, in DBA/2 mice, the numbers of CD45^+^ immune cells and neutrophils were higher in reinfected mice than in primary-infected mice. Thus, in the present study, we demonstrated that the DBA/2 mouse strain was most susceptible to Mp infection, suggesting that it could be an appropriate strain to examine the effects of Mp infection. Furthermore, our findings revealed that simultaneous examination and comparison of various inbred mouse strains could be a useful means of elucidating the pathogenesis of Mycoplasma pneumonia toward the development of efficacious therapeutics against Mp infection.

## Materials and Methods

### Mice

In this study, 6- to 7-week-old, male A/J, BALB/c, C3H/HeJ, C3H/HeN, C57BL/6J, CBA/N, DBA/1, and DBA/2 mice were purchased from SLC (Hamamatsu, Japan). All mice were housed under a 12:12-h light/dark cycle (lights on, 8:00 am), with unrestricted access to food and water. Animal experiments were conducted according to Osaka University’s institutional guidelines for the ethical treatment of animals and were approved by the Animal Care and Use Committee of the Research Institute for Microbial Diseases, Osaka University, Japan (protocol number, BIKEN-AP-H29-01-1).

### Culture Condition of Mycoplasma pneumoniae

Mp (strain: FH) was purchased from the American Type Culture Collection (Manassas, VA, USA). Mp was cultured as described previously (Tamiya et al., 2020). Mp in PPLO medium at 2 × 10^9^ colony-forming units (CFU)/ml was used.

### *Mycoplasma pneumoniae* Infection

Briefly, anesthetized mice were intranasally infected with 6.0 × 10^7^ CFU of Mp in 40 μl phosphate-buffered saline (PBS). On days 1, 3, and 5 post-infection, the Mp titer, lactate dehydrogenase (LDH) activity, immune cell numbers, and cytokine concentrations were analyzed in BALF and recovered by lavaging the lung with 1.2 ml PBS.

### Quantification of *Mycoplasma pneumoniae* by Bacterial Culture

The Mp load in BALF was analyzed by using real-time polymerase chain reaction (real-time PCR) as described previously (Tamiya et al., 2020). To measure the Mp bacterial load by Mp culture, BALF was serially diluted and cultured on a PPLO agar plate, which contained PPLO agar (Difco Laboratories), 10% horse serum (Thermo Fisher Scientific), 0.25% yeast extract (Difco Laboratories), 1000 U/ml penicillin G, and 0.25% glucose. After incubation at 37°C for 1 to 2 weeks, colonies were counted using a dissecting microscope.

### Immune Cell Analysis of Bronchoalveolar Lavage Fluid

The number of immune cells in the BALF was determined as previously described (Tamiya et al., 2020). Briefly, the cell suspension in BALF was analyzed by flow cytometry (NovoCyte Flow Cytometer, ACEA Bioscience, San Diego, CA, USA). We defined neutrophils as CD45^+^ Ly6G^+^ CD11b^+^ Siglec-F^−^ cells, and alveolar macrophages as CD45^+^ Ly6G^−^ CD11c^+^ Siglec-F^+^ cells.

### Cytokine Assay and Lactate Dehydrogenase Assay

After Mp infection, concentrations of interleukin (IL)-1α, IL-6, and IL-12 p40 in BALF were determined using the appropriate enzyme-linked immunosorbent assay (ELISA) kit (BioLegend) in accordance with the manufacturer’s instructions. The level of LDH activity in BALF was determined using the Cytotoxicity LDH Assay Kit-WST (Dojindo, Kumamoto, Japan) in accordance with the manufacturer’s instructions.

### Recombinant P1 Protein

The amino acid sequence for P1 was derived from the FH strain. cDNA of P1 corresponding to the amino acid residues 60 to 1525, with an N-terminal hexahistidine tag was cloned into a pCold I vector (TAKARA BIO, Shiga, Japan). Recombinant P1 protein was expressed using *E. coli* as described previously with small modifications (Kenri et al., 2019). Briefly, after incubation of *E. coli* at 15°C, the recombinant P1 was purified using a Ni-Sepharose 6 Fast Flow column (GE Healthcare, Diegem, Belgium).

### Immune Responses to *Mycoplasma pneumoniae*

Briefly, anesthetized mice were intranasally infected with 6.0 × 10^7^ CFU of Mp in 40 μl of PBS. Control mice were treated with 40 μl of PBS, intranasally administered under anesthesia. On day 14, plasma and spleen samples were collected and analyzed as follows. The plasma levels of Mp- and P1-specific IgG were determined using ELISA as described previously (Tamiya et al., 2020). To detect P1-specific total IgG, ELISA plates (Corning, Corning, NY, USA) were coated overnight at 4°C with recombinant P1 (1 μg/ml) in PBS. To assess cytokine production by splenocytes, pooled splenocytes from each mouse (1 × 10^6^ cells per well) were stimulated for 3 days at 37°C with Mp (5 × 10^7^ CFU/ml) or left unstimulated. After incubation, the concentrations of interferon (IFN)-*γ* and IL-17A were determined in culture supernatants by ELISA (BioLegend) in accordance with the manufacturer’s instructions.

### Reinfection of *Mycoplasma pneumoniae*

Briefly, anesthetized mice were intranasally infected with 6.0 × 10^7^ CFU of Mp in 40 μl of PBS. On day 14 post-infection, mice were intranasally reinfected with 6.0 × 10^7^ CFU Mp in 40 μl of PBS under anesthesia. The day following Mp reinfection, the Mp titer and the number of immune cells in BALF were determined as previously described.

### CD4^+^ T-Cell Depletion

Briefly, anesthetized mice were intranasally infected with 6.0 × 10^7^ CFU of Mp in 40 μl of PBS. On day 13 post-infection, 100 μg/mouse anti-CD4 antibody (clone: GK1.5) or 100 μg/mouse isotype-control antibody (clone: RTK4530) was intraperitoneally injected into mice, followed by reinfection intranasally on day 14 using 6.0 × 10^7^ CFU Mp in 40 μl of PBS under anesthesia. On day 15 post-infection, BALF was collected, and the number of immune cells was determined as previously described.

### Statistical Analyses

Statistical analyses were performed using GraphPad Prism 7J (GraphPad Software, San Diego, CA, USA). In the graphs, values are presented as mean ± standard deviation (SD). Significant differences were detected using Tukey’s test or Student’s *t*-test. A *P*-value <0.05 was considered to statistically significant.

## Results

### *Mycoplasma pneumoniae* Bacterial Load in Bronchoalveolar Lavage Fluid After *Mycoplasma pneumoniae* Infection

**Figure 1:**
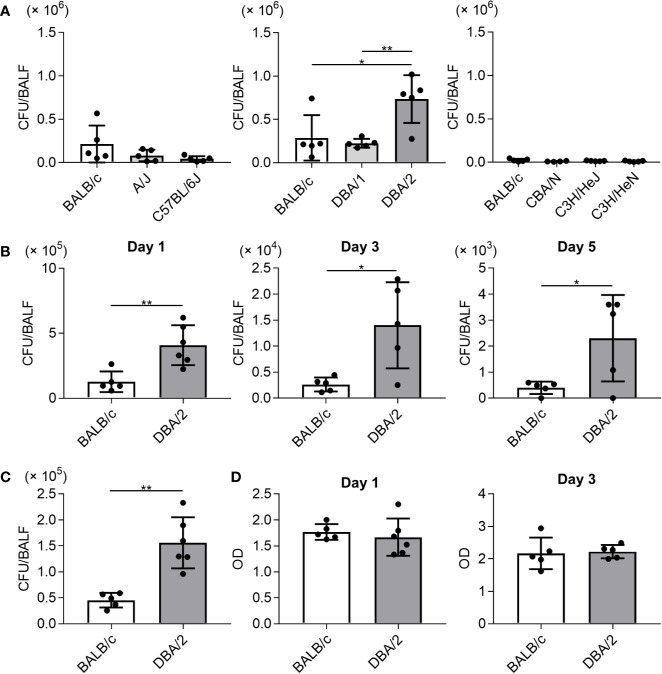
*Mycoplasma pneumoniae* bacterial load in bronchoalveolar lavage fluid after infection in several inbred mouse strains. Mice were infected with *Mycoplasma pneumoniae* (Mp) intranasally. **(A)** On day 1 after infection, the Mp bacterial load in bronchoalveolar lavage fluid (BALF) was determined using real-time PCR. **(B)** On days 1, 3, and 5 post-infection, the Mp bacterial load in BALF obtained from BALB/c and DBA/2 mice was determined by real-time PCR. **(C)** On day 1 post-infection, Mp bacterial load in BALF from BALB/c and DBA/2 mice was analyzed by bacterial culture assay. **(D)** On days 1 and 3 post-infection, the level of lactate dehydrogenase activity in BALF was determined. Data are shown as means ± SD. Each experiment was performed twice. **(A)** *n* = 4 or 5 per group, **(B–D)** *n* = 5 or 6 per group. **(A)** *P < 0.05; **P < 0.01 as indicated by Tukey’s test. **(B, C)** *P < 0.05; **P < 0.01 as indicated by Student’s *t*-test.

To examine susceptibility to Mp infection among different inbred mouse strains, the Mp bacterial load in the lung post-infection was examined. On day 1 after naive mice were intranasally infected with Mp, we measured the Mp bacterial load in BALF by real-time PCR (**Figure 1A**). The bacterial load in BALF from DBA/2 mice, which is a complement component 5 (C5)-deficient strain, was significantly higher than that observed in BALB/c and DBA/1 mice. No other strains demonstrated a higher bacterial load when compared with that observed in BALB/c mice. Interestingly, the bacterial load in A/J mice, another complement C5-deficient strain, was comparable with that observed in BALB/c mice. Further examination of DBA/2 and BALB/c mice revealed that the bacterial load in DBA/2 mice remained higher than that in BALB/c mice on days 3 and 5 post-infection (**Figure 1B**). Using a bacterial culture assay sensitive only to viable Mp, we observed more viable Mp in DBA/2 mice than in BALB/c mice on day 1 post-infection (**Figure 1C**). In addition, we did not observe any difference in the level of LDH activity, as a marker of tissue injury, in BALF on days 1 and 3 post-infection between the BALB/c and DBA/2 mice (**Figure 1D**). Collectively, these results indicate that Mp can better persist in the lungs of DBA/2 mice among the mouse strains evaluated.

### Immune Cell Numbers and Cytokine Levels in Bronchoalveolar Lavage Fluid

**Figure 2:**
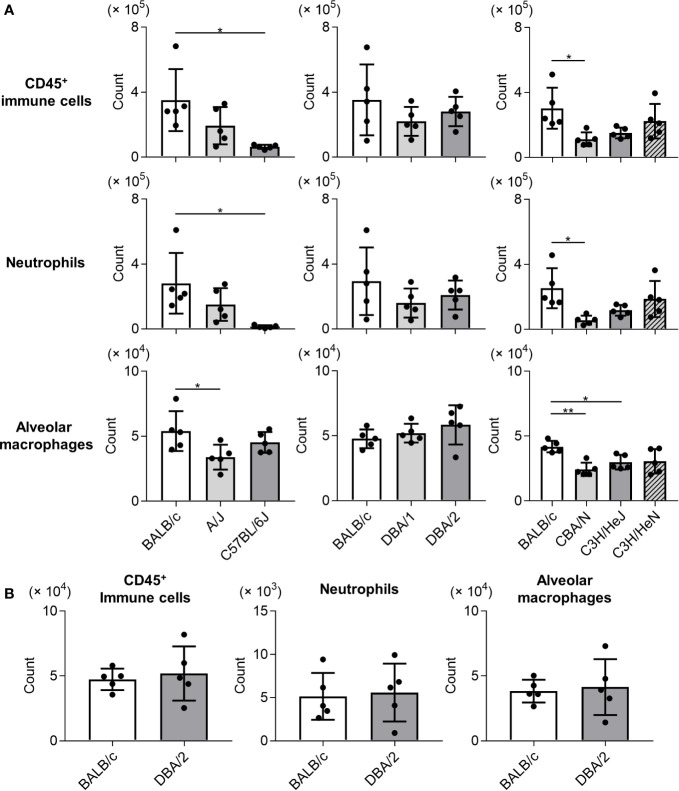
Immune cell numbers in bronchoalveolar lavage fluid after *Mycoplasma pneumoniae* infection in several inbred mouse strains. **(A)** Mice from several strains were infected intranasally with *Mycoplasma pneumoniae*. On day 1 post-infection, the numbers of CD45^+^ immune cells, neutrophils, and alveolar macrophages in bronchoalveolar lavage fluid (BALF) were determined using flow cytometry. **(B)** The numbers of CD45^+^ immune cells, neutrophils, and alveolar macrophages in BALF from naive BALB/c mice and naive DBA/2 mice were determined by flow cytometry. Data are shown as means ± SD. Each experiment was performed twice. **(A, B)** *n* = 5 per group. **(A)** *P < 0.05; **P < 0.01 as indicated by Tukey’s test.

To examine the immune responses of mouse strains to Mp infection, we compared the numbers of immune cells (CD45^+^ cells), neutrophils, and alveolar macrophages in BALF collected on day 1 after Mp infection (**Figure 2A**). The numbers of CD45^+^ immune cells and neutrophils were lower in C57BL/6J and CBA/N mice than in BALB/c mice. Similarly, the number of alveolar macrophages in A/J, CBA/N, and C3H/HeJ mice was lower than those observed in BALB/c mice. In contrast, no significant differences were observed in the number of immune cells, neutrophils, and alveolar macrophages between BALB/c mice and DBA/2 mice, although DBA/2 mice revealed a high Mp bacterial load in the previous experiment (**Figure 1**). In addition, these cell numbers did not differ between naive BALB/c and naive DBA/2 mice (**Figure 2B**).

**Figure 3:**
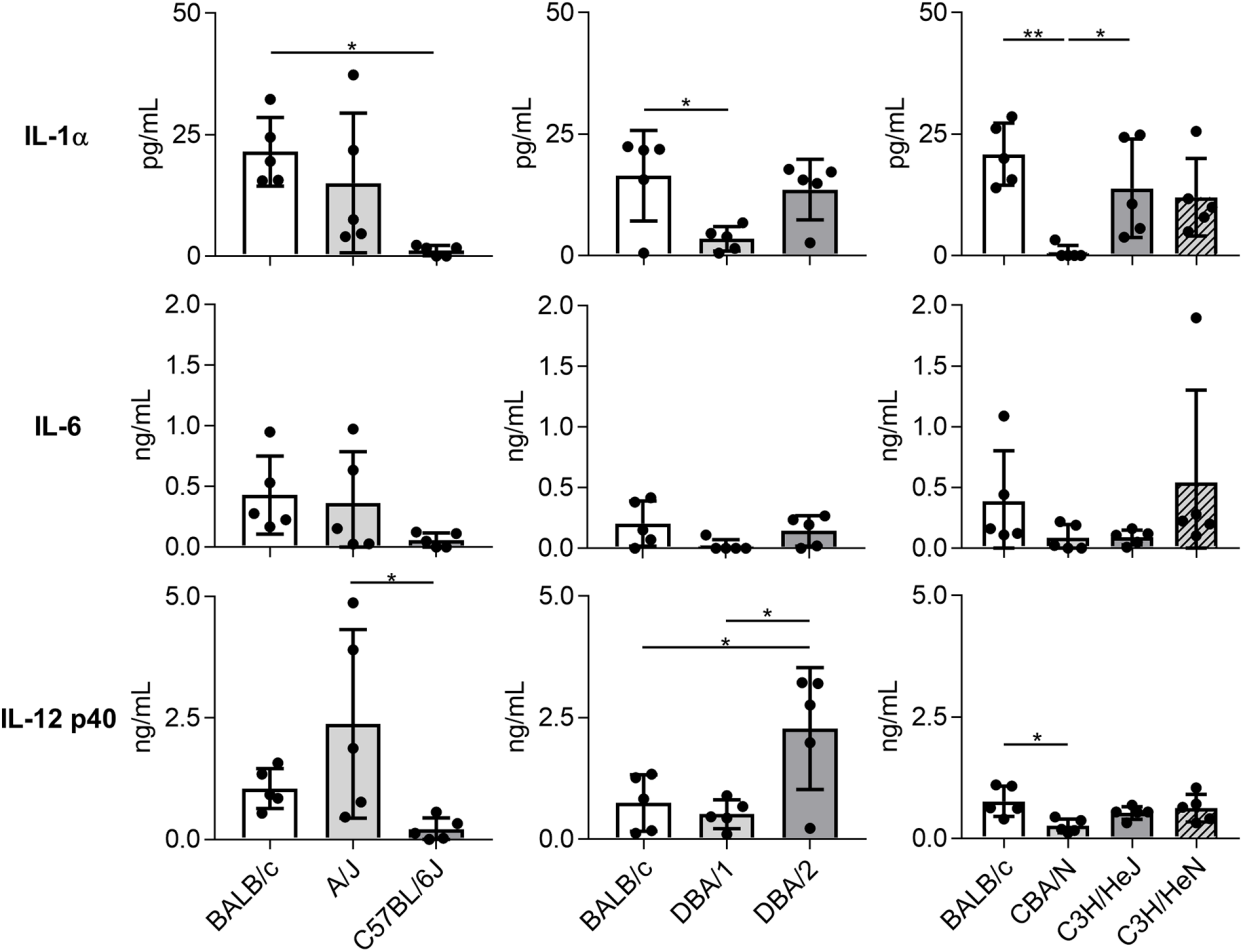
Cytokine levels in bronchoalveolar lavage fluid after *Mycoplasma pneumoniae* infection in several inbred mouse strains. Mice from several strains were infected intranasally with *Mycoplasma pneumoniae*. On day 1 post-infection, the concentrations of cytokines in bronchoalveolar lavage fluid were determined by ELISA. Data are shown as means ± SD. Each experiment was performed twice. *n* = 5 per group. *P < 0.05; **P < 0.01 as indicated by Tukey’s test.

Consistent with results regarding immune cell and neutrophil numbers, the concentrations of IL-1α, IL-6, and IL-12 subunit p40 in BALF on day 1 post-Mp infection were significantly lower or tended to be lower in C57BL/6J, DBA/1, and CBA/N mice than in BALB/c mice (**Figure 3**). In contrast, DBA/2 mice showed a higher level of IL-12 p40 than BALB/c mice, although no significant difference was observed in the IL-1α and IL-6 levels between these two strains. Furthermore, the level of IL-12 p40 in A/J mice tended to be higher than that observed in BALB/c mice. Collectively, these results suggest that mouse strains demonstrate different immune responses to Mp infection.

### *Mycoplasma pneumoniae*-Specific Immune Responses After *Mycoplasma pneumoniae* Infection

**Figure 4:**
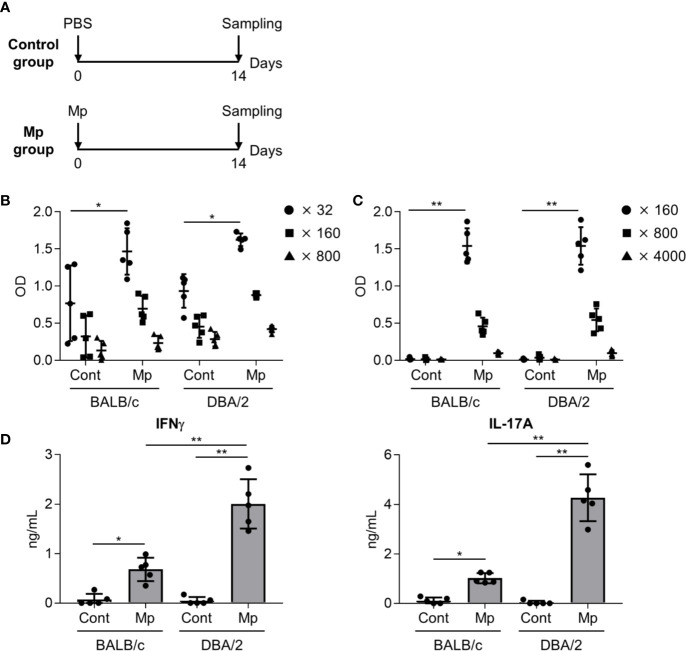
Immune responses after *Mycoplasma pneumoniae* infection. **(A)** Schematic representation of the experiment. **(B–D)** BALB/c and DBA/2 mice were infected intranasally with *Mycoplasma pneumoniae* (Mp). **(B, C)** On day 14 post-infection, the levels of **(B)** Mp-specific IgG in 32-, 160-, and 800-fold diluted plasma, and **(C)** recombinant P1-specific IgG in 160-, 800-, and 4000-fold diluted plasma were determined using ELISA. **(D)** On day 14 post-infection, splenocytes were cultured with Mp for 3 days and the levels of IFN-*γ* and IL-17A in the culture supernatant were determined by ELISA. Data are shown as means ± SD. Each experiment was performed twice. **(B–D)** *n* = 5 per group. **(B, C)** Significant differences were observed only in the **(B)** 32-fold– and **(C)** 160-fold–diluted plasma samples. **(B–D)** *P < 0.05; **P < 0.01 as indicated by Tukey’s test.

Next, we examined the adaptive immune response after Mp infection in BALB/c and DBA/2 mice as IL-12 p40, the level of which differed significantly between the two strains in the previous experiment (**Figure 3**), plays a crucial role in the induction of adaptive immune responses (Croxford et al., 2014). On day 14 post-Mp infection, Mp-specific antibody responses and T-cell responses were analyzed (**Figure 4A**). In BALB/c and DBA/2 mice, the plasma levels of Mp- and P1-specific IgG were increased following Mp infection although the background level of Mp-specific IgG in naive BALB/c and naive DBA/2 mice was already high (**Figures 4B, C**). Moreover, no significant difference was observed in the plasma levels of Mp- and P1-specific IgG after Mp infection, between BALB/c and DBA/2 mice. Additionally, we examined the Mp-specific CD4^+^ T-cell response in mice after Mp infection by examining cytokine production in splenocytes (**Figure 4D**). In contrast to the antibody responses, the levels of IFN-*γ* (a Th1-related cytokine) and IL-17A (a Th17-related cytokine) produced by splenocytes after Mp stimulation were significantly higher in Mp-infected DBA/2 mice than in Mp-infected BALB/c mice. Overall, these results suggest that Mp infections induce stronger Mp-specific Th1 and Th17 responses in DBA/2 mice than in BALB/c mice.

**Figure 5:**
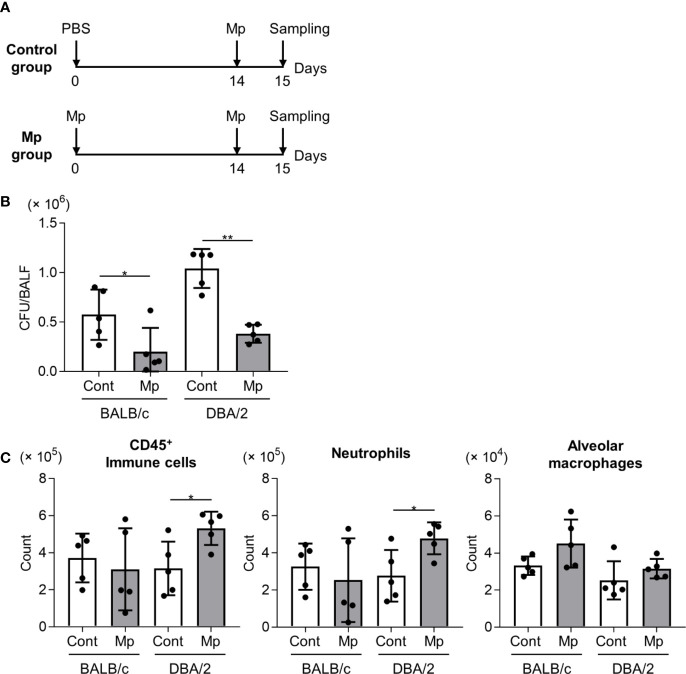
*Mycoplasma pneumoniae* bacterial load and immune cell numbers in bronchoalveolar lavage fluid after *Mycoplasma pneumoniae* reinfection in BALB/c and DBA/2 mice. **(A)** Schematic representation of the experiment. **(B, C)** BALB/c mice and DBA/2 mice were infected intranasally with *Mycoplasma pneumoniae* (Mp). On day 14 post-infection, mice were intranasally reinfected with Mp. On the day after reinfection, **(B)** Mp bacterial load and **(C)** the numbers of CD45^+^ immune cells, neutrophils, and alveolar macrophages in bronchoalveolar lavage fluid were determined by **(B)** real-time PCR and **(C)** flow cytometry. Data are shown as means ± SD. Each experiment was performed twice. **(B, C)** *n* = 5 per group. *P < 0.05; **P < 0.01 as indicated by Student’s *t*-test.

To evaluate the effects of the robust Mp-specific Th1 and Th17 responses observed in DBA/2 mice following Mp reinfection, BALB/c and DBA/2 mice were intranasally reinfected with Mp on day 14 after the initial Mp infection (**Figure 5A**). In both BALB/c and DBA/2 mice, the prior Mp infection significantly lowered the bacterial load in BALF after reinfection (**Figure 5B**). Additionally, the number of CD45^+^ immune cells and neutrophils was significantly higher in the reinfected mice than in the primary-infected mice in DBA/2 but not BALB/c mice (**Figure 5C**). The number of alveolar macrophages remained comparable between the two strains.

**Figure 6:**
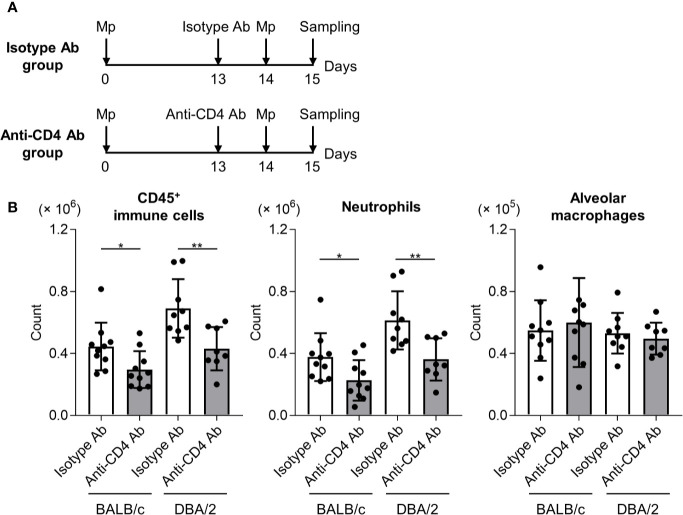
CD4^+^ T cell-dependent neutrophil infiltration in reinfected mice. **(A)** Schematic representation of the experiment. **(B)** BALB/c and DBA/2 mice were infected intranasally with *Mycoplasma pneumoniae* (Mp). On day 13 post-infection, mice were injected intraperitoneally with anti-CD4 or isotype-control antibody. On day 14, mice were intranasally reinfected with Mp. The day after Mp reinfection, the numbers of CD45^+^ immune cells, neutrophils, and alveolar macrophages in bronchoalveolar lavage fluid were determined by flow cytometry. Data are shown as means ± SD. Each experiment was performed twice. *n* = 8–10 per group. *P < 0.05; **P < 0.01 as indicated by Student’s *t*-test.

To examine the effects of T cell responses to neutrophil infiltration after Mp reinfection in DBA/2 mice, an anti-CD4 antibody was used to deplete CD4^+^ T cells in primary-infected mice before Mp reinfection. After Mp reinfection, we evaluated lung inflammation (**Figure 6A**). In both BALB/c mice and DBA/2 mice, the numbers of immune cells and neutrophils, but not alveolar macrophages, after Mp reinfection were significantly decreased in mice treated with the anti-CD4 antibody when compared with the isotype-control antibody (**Figure 6B**). These data suggest that enhanced Th1 and Th17 responses are key factors in the increased neutrophil lung infiltration observed after Mp reinfection in DBA/2 mice.

## Discussion

Here, we found that the bacterial load after Mp infection was highest in DBA/2 mice (**Figure 1**). Furthermore, we observed that the bacterial load in A/J mice after Mp infection was comparable to that in BALB/c mice (**Figure 1A**), which is interesting as DBA/2 mice and A/J mice are both C5 deficient (Wetsel et al., 1990). Thus, a disrupted complement cascade is insufficient to produce a higher bacterial load in the DBA/2 mice. Several studies have shown that DBA/2 mice are susceptible to infection by other pathogens such as *Pseudomonas aeruginosa* and *Bacillus anthracis*, although the precise mechanisms underlying this susceptibility remain unclear (Gosselin et al., 1995; Wilson et al., 2007; Yadav et al., 2011). In addition, Wilson *et al*. have shown that macrophages from DBA/2 mice have lower bactericidal activity than those from C57BL/6 mice, although the macrophages from DBA/2 mice retain their bacterial phagocytic ability (Wilson et al., 2007). Together with the findings of Lai et al. (2010), alveolar macrophages, which are specialized macrophages located in the alveolar region of the lower respiratory tract (Hussell and Bell, 2014), are crucial for the removal of Mp from the lungs (Lai et al., 2010). The high bacterial load observed in DBA/2 mice may be a result of reduced macrophage bactericidal activity or a combination of reduced macrophage bactericidal activity and the C5 deficiency. Moreover, there may be other factors present in DBA/2 mice that produce a suitable environment for Mp survival in lung epithelial cells. Therefore, further investigation is needed to elucidate the underlying cause of the observed high Mp bacterial load in this mouse strain.

Among the mouse strains, we observed different patterns of infiltrated immune cells in the lung (**Figure 2A**). The numbers of CD45^+^ immune cells and neutrophils in C57BL/6J and CBA/N mice were significantly lower than those in BALB/c mice (**Figure 2A**). Additionally, consistent with the results for immune cell infiltration, the levels of cytokines in BALF from C57BL/6J and CBA/N mice were significantly lower or tended to be lower than those in BALB/c mice (**Figure 3**). The production of these cytokines is mainly dependent on TLR2 signaling (Shimizu, 2016). Liu *et al*. have shown that dendritic cells from BALB/c mice highly express mRNAs of TLRs, including TLR2, when compared with dendritic cells from C57BL/6 mice (Liu et al., 2002), indicating the possibility that the strength of TLR2 signaling might differ from strain to strain. Thus, a difference in TLR2 expression might underlie the observed differences in cytokine production and neutrophil infiltration in C57BL/6J and CBA/N mice.

The complement system is the first line of immune defense. Complement components bind to foreign substances such as microbes to mark them for clearance from the body. Furthermore, activation of the complement cascade induces the production of anaphylatoxins such as C3a and C5a, which activate various cell types, including mast cells, macrophages, and granulocytes (Wood et al., 2018). Among the various anaphylatoxins, C5a promotes inflammation by inducing neutrophil infiltration and activation *via* the C5a receptor on the neutrophil surface (Wood et al., 2018). Inhibition of the C5a receptor suppresses acute systemic inflammation (Czermak et al., 1999; Rittirsch et al., 2008). Here, we observed no significant differences in the numbers of CD45^+^ immune cells and neutrophils between BALB/c and DBA/2 mice and between BALB/c and A/J mice after Mp infection (**Figure 2A**), suggesting that since both DBA/2 and A/J strains are C5-deficient, neutrophil infiltration after Mp infection is not mediated by C5a. In addition, considering the result of the LDH activity in BALF (**Figure 1D**), lung-tissue damage that resulted from neutrophil infiltration in DBA/2 mice was not aggravated compared with that in BALB/c mice.

However, we found that the level of IL-12 p40 was significantly higher in DBA/2 mice than that in BALB/c mice and that the level of IL-12 p40 tended to be higher in A/J mice than in BALB/c mice (**Figure 3**). Moreover, we observed that Mp-specific Th1 and Th17 responses were greater in DBA/2 mice than in BALB/c mice (**Figure 4D**). IL-12 p40 is a subunit of IL-12, which contributes to the differentiation of Th1 cells from naive T cells, but also of IL-23, which plays an important role in the differentiation of Th17 cells (Croxford et al., 2014). Consequently, the enhanced Th1 and Th17 responses observed in DBA/2 mice after Mp infection may be caused by the enhanced production of IL-12 p40. Furthermore, recent reports have shown that C5a exerts anti-inflammatory effects by suppressing the production of IL-17A and IL-23 during endotoxic shock in mice (Bosmann et al., 2012). Additionally, Lajoie *et al*. have reported that C5a suppresses IL-23–mediated Th17 responses in mice (Lajoie et al., 2010). These findings suggest that the C5 deficiency in DBA/2 mice could increase IL-12 p40 production, which is consistent with the enhanced Th1 and Th17 responses.

In our reinfection experiment, we found that prior Mp infection resulted in increased infiltration of CD45^+^ immune cells and neutrophils into BALF after subsequent reinfection in DBA/2 mice but not in BALB/c mice (**Figure 5**). In a previous report, we revealed that CD4^+^ T cells do not induce neutrophil infiltration after Mp infection in naive mice (Tamiya et al., 2020). Collectively, these data suggest that Mp-specific Th1 and/or Th17 cells induced by prior Mp infection were the major effector cells for the enhanced neutrophil infiltration observed in DBA/2 mice after Mp reinfection. These findings are consistent with previous reports showing that natural reinfection by Mp leads to the exacerbation of pneumonia in mice and hamsters (Cimolai et al., 1995; Chu et al., 2006). Thus, prior sensitization to Mp likely results in immune responses that induce greater inflammation after subsequent Mp reinfection when compared with that after primary infection.

Indeed, Mp-specific CD4^+^ T cell responses are important for exacerbated inflammation (Dobbs et al., 2009). For example, Yang *et al*. have shown that an elevated Th1/Th2 ratio is associated with disease severity in mycoplasma pneumonia in humans (Yang et al., 2019). Other reports have shown increased levels of IFN-γ in the serum of children with mycoplasma pneumonia (Fan et al., 2015) or refractory mycoplasma pneumonia (Wang et al., 2014; Zhang et al., 2016b). However, some reports imply that IFN-γ serum levels are not related to the severity of Mp infection (Koh et al., 2001; Fan et al., 2016), although the reason for these contradictory clinical findings remains unclear. Furthermore, significantly higher frequencies of Th17 cells, which induce neutrophil infiltration into sites of inflammation *via* the production of IL-17 (Way et al., 2013), and higher levels of IL-17A have been observed in patients with refractory mycoplasma pneumonia (Wang et al., 2016; Zhao et al., 2020). The present results suggest that DBA/2 mice demonstrate CD4^+^ T cell responses to Mp infection that are similar to those observed in humans. Thus, further investigations are needed to characterize the type of CD4^+^ T-cells, which is responsible for neutrophil infiltration induced by prior infection of Mp in DBA/2 mice.

In summary, we revealed that DBA/2 mice could be useful for examining the effects of Mp infection and that comparison of strain-related differences in response to Mp infection could be a beneficial strategy for elucidating the pathogenesis of Mycoplasma pneumonia.

## Data Availability Statement

The original contributions presented in the study are included in the article/supplementary materials; further inquiries can be directed to the corresponding author.

## Ethics Statement

The animal study was reviewed and approved by The Animal Care and Use Committee of the Research Institute for Microbial Diseases, Osaka University, Japan.

## Author Contributions

ST and YY designed the experiments and interpreted the results. ST and EY performed the experiments and collected and analyzed the data. KS provided technical support and conceptual advice. ST and YY wrote the manuscript. All authors contributed to the article and approved the submitted version.

## Funding

This study was supported by grants from the Japan Society for the Promotion of Science (JSPS KAKENHI Grant Numbers JP17H04009, JP18K19401, and JP20H03404 to YY) and a Nagai Memorial Research Scholarship from the Pharmaceutical Society of Japan (to ST).

## Conflict of Interest

KS and YY are employed by the Research Foundation for Microbial Diseases of Osaka University.

The remaining authors declare that the research was conducted in the absence of any commercial or financial relationships that could be construed as a potential conflict of interest.
